# Supplementary material for: Unveiling Species Diversity Within Early-Diverging Fungi from China IX: Four New Species of Mucor (Mucoromycota)
Source: J Fungi (Basel). 2025 Sep 19;11(9):682. doi: 10.3390/jof11090682 (PMC12470528; doi:10.3390/jof11090682)
Supplement: Supplementary file 1 [file jof-11-00682-s001.zip › Table S1.pdf]

Table S1. GenBank accession numbers of *Mucor* and *Backusella* strains in this study.

Species	Strains	GenBank accession numbers		
		ITS	LSU	*RPB1*
M. abundans	CBS 521.66	JN206110	JN206457	NA
M. abundans	CBS 388.35*	JN206111	NG_063979	MT500100
M. albicolonia	CNUFC CY2027*	PP844894	PP851426	NA
M. albicolonia	CNUFC CY2028	PP844895	PP851427	NA
M. albicolonia	CNUFC CY2311	PP844896	PP851428	NA
M. aligarensis	CBS 993.70*	NR_103634	NG_057920	MT500089
M. atramentarius	XY07583	OL620123	NA	NA
M. aseptatophorus	MFLU 21–0040*	MZ433252	MZ433249	NA
M. atramentarius	CBS 202.28*	JN205994	JN206418	MF495236
M. atramentarius	XY06751	OL620124	NA	NA
M. bacilliformis	CBS 251.53*	JN206083	JN206451	MT500067
M. bacilliformis	CBS 573.70	JN206084	JN206452	NA
M. breviphorus	CGMCC 3.16135*	OL678183	NA	NA
M. cheongyangensis	CNUFC ICL1*	MN592639	MN592643	NA
M. cheongyangensis	CNUFC ICL2	MN592640	MN592644	NA
M. chuxiongensis	NYNU 174111*	NR_185548	NG_228784	NA
M. cryophilus	CNUFC CHS1*	PP844923	PP852708	PP886119
M. donglingensis	CGMCC 3.16139*	OL678190	NA	NA
M. donglingensis	XY08501	OL678191	NA	NA
M. durus	CBS 156.51*	JN206112	JN206456	MT500101
M. endophyticus	CBS 385.95*	JN206159	JN206448	MT500068
M. flavus	CBS 230.35	JN206061	JN206464	MT500088
M. flavus	CBS 234.35*	JN206051	JN206468	MT500091
M. flavus	CBS 126.70	JN206049	JN206469	MT500092
M. fusiformisporus	CGMCC 3.16141*	OL678194	NA	NA
M. fusiformisporus	XY08153	OL678195	NA	NA
M. fusiformisporus	XY08154	OL678196	NA	NA
M. fusiformisporus	XY08117	OL678197	NA	NA
M. gigasporus	CBS 566.91*	JN206247	JN206494	NA
M. gigasporus	CBS 383.95	JN206246	NA	NA
M. glutinatus	CNUFC CY2012*	PP844899	PP852710	PP886121
M. glutinatus	CNUFC CY2016	PP844900	PP852711	PP886122
M. guilliermondii	CBS 174.27*	JN206082	JN206475	MT5000.64
M. globosporus	**CGMCC 3.28970***	**PV819211**	**PV833754**	**PX048331**
M. globosporus	**XG09770-10-2**	**PV819212**	**PV833755**	**PX048332**
M. hemisphaericum	CGMCC 3.16143*	OL678200	NA	NA
M. heterogamus	CBS 405.58*	JN206167	JN206487	NA
M. heterogamus	CBS 338.74	JN206169	JN206488	NA
M. hiemalis	CBS 201.65*	JN206125	HM849683	MT500073
M. homothallicus	CGMCC 3.16144*	OL678201	NA	NA

M. homothallicus	XY06967	OL678202	NA	NA
M. irregularis	CBS 700.71*	JN206154	JN206450	MT500065
M. irregularis	CBS 103.93	JN206150	HM849684	NA
M. multimorphus	**CGMCC 3.28968***	**PV819207**	**PV833750**	**PV889321**
M. multimorphus	**XG00398-8-2**	**PV819208**	**PV833751**	**PV889322**
M. japonicus	CBS 154.69	JN206158	JN206446	MT500071
M. lobatus	CGMCC 3.16146*	OL678204	NA	NA
M. lobatus	XY07029	OL678205	NA	NA
M. luteus	CBS 243.35*	NR_120224	HM849685	JX976289
M. merdicola	URM 7222*	KT960374	KT960372	MT500070
M. minutus	CBS 586.67	JN206048	JN206463	MT500085
M. moelleri	CBS 444.65	JN206114	HM849682	MT500098
M. moniliformis	CGMCC 3.16147*	OL678206	NA	NA
M. moniliformis	XY07458	OL678207	NA	NA
M. mucedo	CBS 836.73	JN206092	NA	NA
M. mucedo	CBS 640.67*	JN206085	HM849687	MT500079
M. nidicola	XY08161	OL620143	NA	NA
M. orantomantidis	CNUFC-MID1-1*	MH594737	MH591457	NA
M. orantomantidis	CNUFC-MID1-2	MH594738	MH591458	NA
M. orientalis	CGMCC 3.16148*	OL678208	NA	NA
M. paraorantomantidis	CNUFC CY205*	PP844901	PP851431	PP893224
M. paraorantomantidis	CNUFC CY206	PP844902	PP851432	PP893225
M. piriformis	CBS 169.25*	JN206028	HM849681	MT500077
M. piriformis	CBS 527.68	JN206034	JN206476	NA
M. plasmaticus	CBS 275.49	JN206078	JN206483	MT500084
M. plasmaticus	CBS 402.73	JN206081	NA	NA
M. polymorphus	**CGMCC 3.28969***	**PV819209**	**PV833752**	**PV948857**
M. polymorphus	**XG09597-11-2**	**PV819210**	**PV833753**	**PV948858**
M. robustus	CGMCC 3.16151*	OL678212	NA	NA
M. robustus	XY08976	OL678213	NA	NA
M. robustus	XY09024	OL678214	NA	NA
M. rongii	CICC 41725*	MK903014	MK903013	MT815281
M. saturninus	CBS 974.68*	JN206072	JN206458	MT500086
M. saturninus	CNUFC IO1	PP843591	PP851433	PP886123
M. silvaticus	CBS 249.35	JN206122	JN206455	NA
M. silvaticus	CBS 412.71*	JN206124	NA	MT500066
M. sino-saturninus	CGMCC 3.16152*	OL678215	NA	NA
M. sp.	CBS 334.71	JN206248	JN206518	MT500056
M. strictus	CBS 100.66	JN206035	JN206477	NA
M. xizangensis	**CGMCC 3.28971***	**PV819213**	**PV833756**	**PV973985**
M. xizangensis	**XG10424-13-2**	**PV819214**	**PV833757**	**PV973986**
M. zonatus	CBS 148.69*	JN206104	JN206454	MT500105
B. lamprospora	CBS 118.08*	JN206268	JN206531	OP832467

Notes: New species discovered herein are shown in bold. The asterisk "*" indicates the ex-type or ex-holotype strains. The "NA" stands for "not available".
